# Supplementary material for: Radiomic Consensus Clustering in Glioblastoma and Association with Gene Expression Profiles
Source: Cancers (Basel). 2024 Dec 21;16(24):4256. doi: 10.3390/cancers16244256 (PMC11674874; doi:10.3390/cancers16244256)
Supplement: Supplementary file 1 [file cancers-16-04256-s001.zip › cancers-3352614-supplementary.pdf]

Supplemental Materials

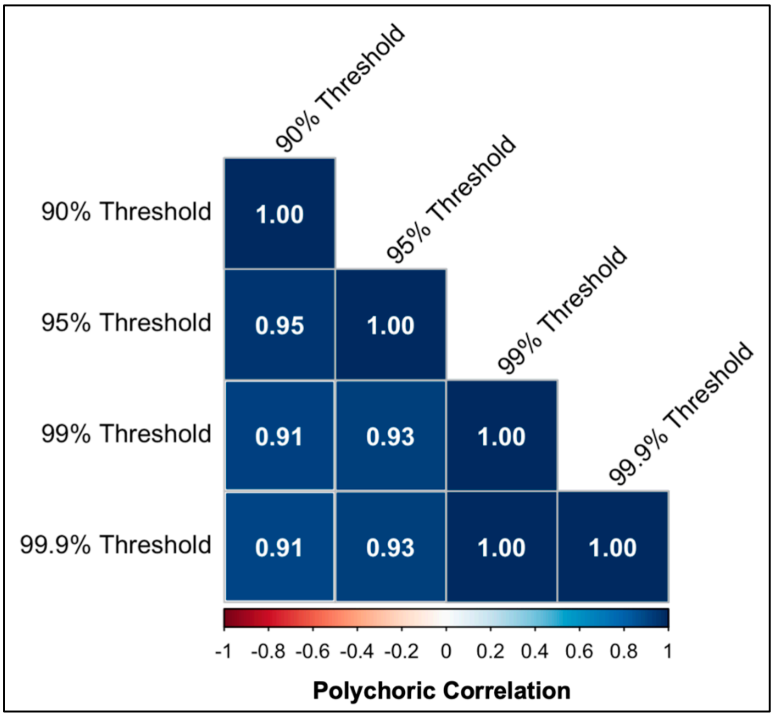

Figure S1. Polychoric correlation of cluster assignments across different absolute pairwise correlation cut-offs for radiomic feature selection process.

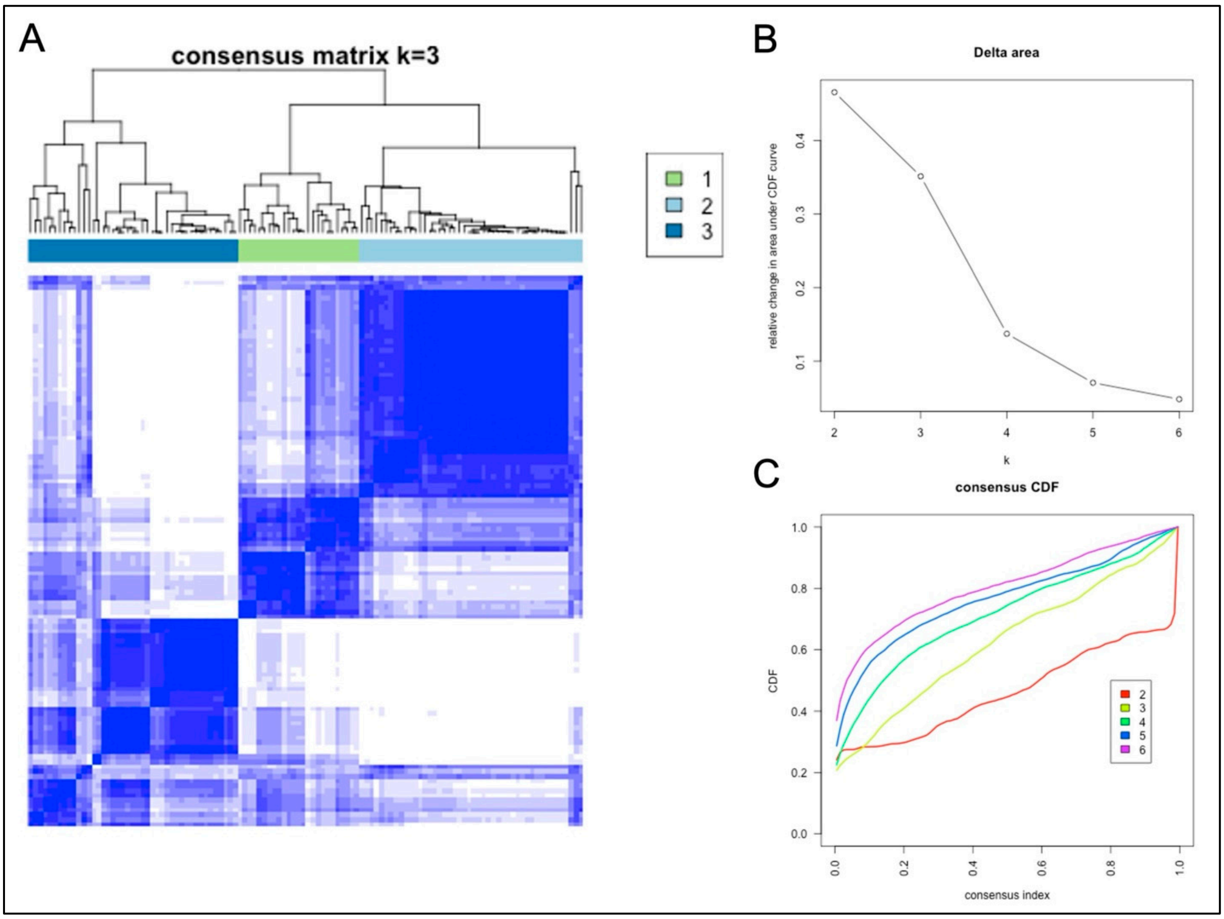

Figure S2. Unsupervised consensus clustering results using radiomic features from the GBM cohort. Includes (A) consensus cluster similarity matrix, (B) consensus CDF delta area plot, and (C) consensus CDF plot.

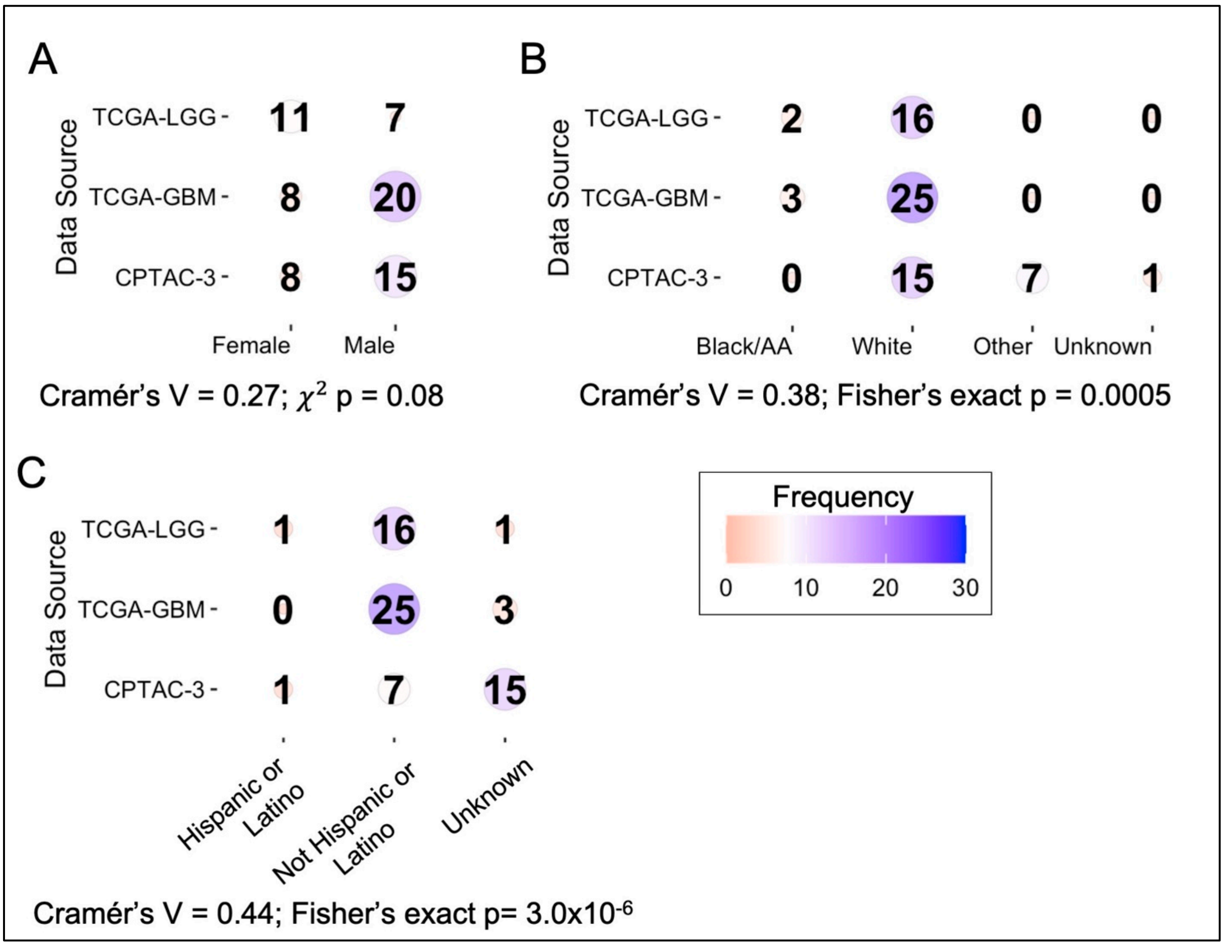

Figure S3. Correlation of demographic variables across data sets (i.e., TGCA-LGG, TCGA-GBM, or CPTAC-3-GBM) displayed as contingency tables. Categorical covariables include (A) patient sex, (B) race group, and (C) ethnicity. Correlation was determined with Cramér's V test and significant difference in counts across data sources was determined with χ^2-test or Fisher's exact test, when appropriate.

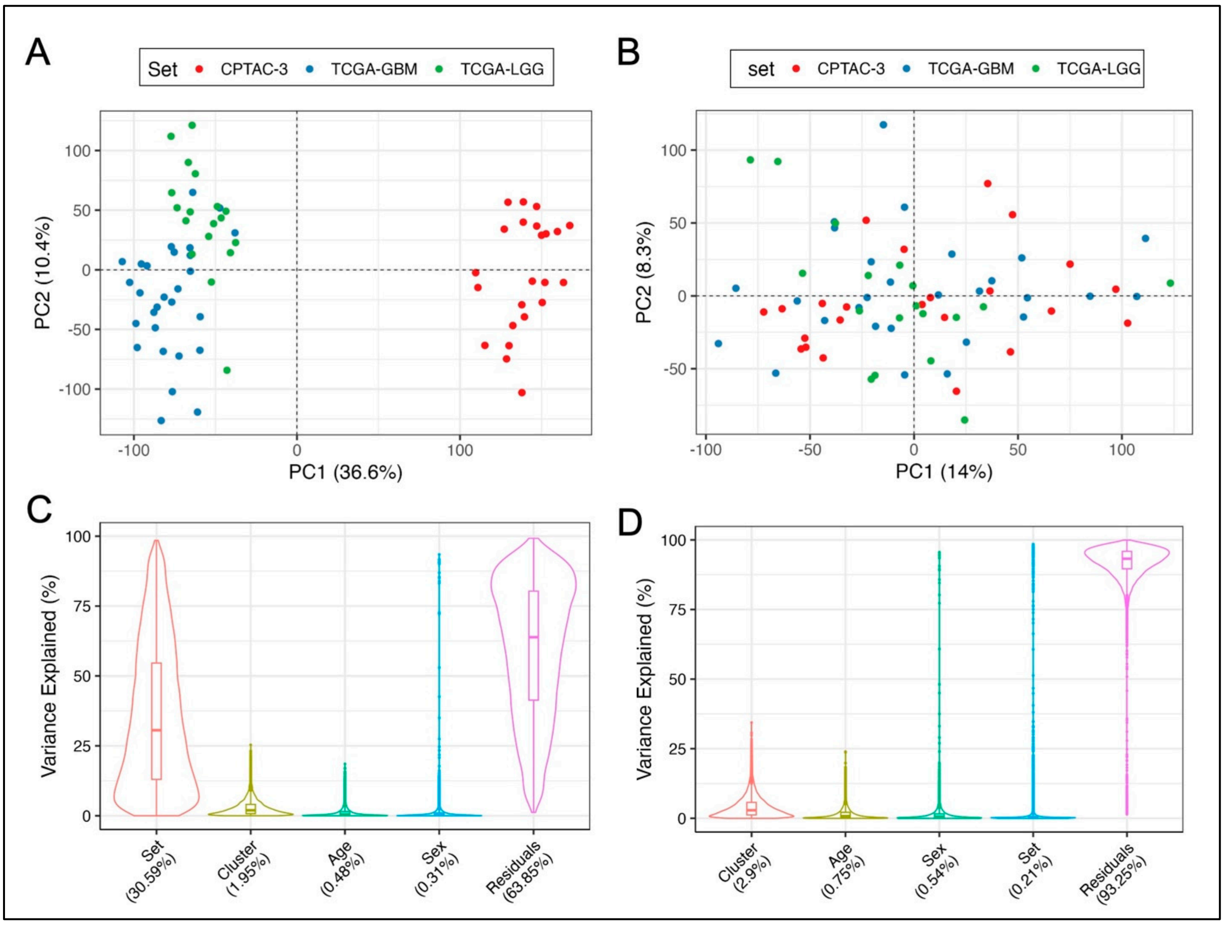

Figure S4. Principal components analyses (A-B) and explained variance plots (C-D) for gene expression data before and after batch effect adjustment using Combat-Seq, respectively. Set represents different data sources (i.e., TGCA-LGG, TCGA-GBM, or CPTAC-3-GBM).

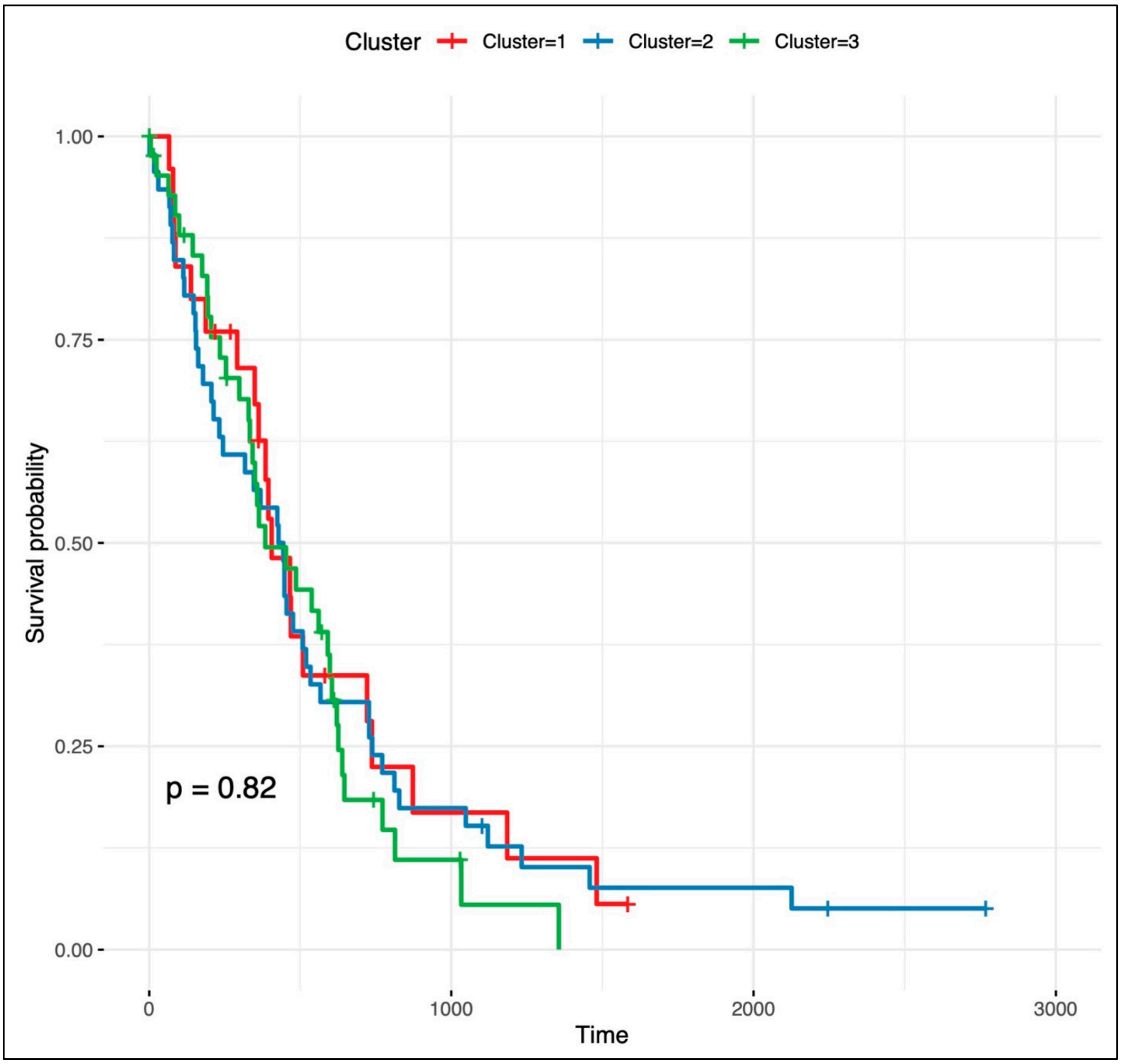

Figure S5. Overall survival probability curves stratified by radiomic clusters. Time represented in days. P value represents log-rank test.

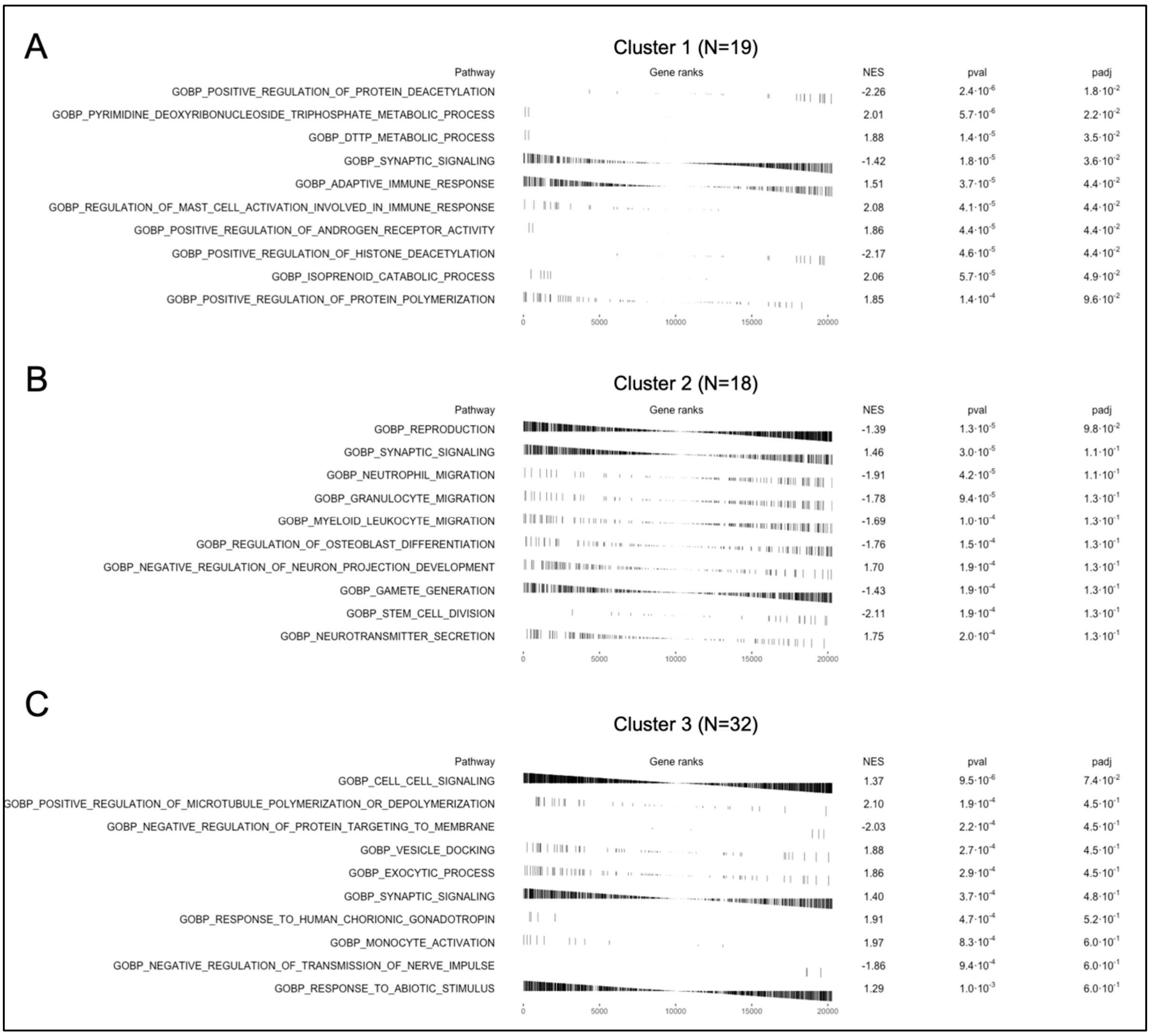

Figure S6. Top ten enriched pathways (sorted by p-adjusted value) from Gene Set Enrichment Analysis. Plot tables are separated for each cluster: (A) Cluster 1, (B) Cluster 2, and (C) Cluster 3.

Table S1. Summary of differentially expressed genes identified in two of the three clusters.
[1] $log_2FC = log_2$ fold-change of gene expression; [2] Bolded lines represent genes significantly enriched in Cluster 1 after correction for multiple comparisons.

Symbol	log_2FC [1]		p		p-adj.	
**Cluster**	**1**	**2**	**1**	**2**	**1**	**2**
H3C8 [2]	**1.23**	**-0.92**	**6.5E-05**	**4.0E-03**	**0.04**	**0.78**
TMEM216	0.67	-0.81	7.5E-04	4.3E-05	0.08	0.56
CPNE8-AS1	1.00	-1.40	8.6E-03	1.9E-04	0.18	0.56
SERINC5	-0.63	0.70	4.3E-03	1.7E-03	0.14	0.78
LINC00632	-1.50	1.56	2.6E-03	2.0E-03	0.12	0.78
PNPLA4	0.92	-0.87	1.4E-03	2.9E-03	0.10	0.78
GPR179	-0.96	0.89	2.6E-03	6.1E-03	0.12	0.78
H4C11	1.09	-0.83	2.6E-04	7.2E-03	0.06	0.78
FBXW4P1	-0.73	0.72	5.4E-03	7.2E-03	0.15	0.78
APBA1	-0.56	0.58	8.7E-03	7.5E-03	0.18	0.78
RGS22	1.16	-1.18	8.0E-03	7.6E-03	0.17	0.78
COL6A6	-0.73	0.71	5.7E-03	7.8E-03	0.15	0.78
PMAIP1	1.09	-0.90	9.4E-04	8.5E-03	0.09	0.78
LINC02831	0.71	-0.71	8.0E-03	9.3E-03	0.17	0.78
**Cluster**	**1**	**3**	**1**	**3**	**1**	**3**
PRX	**-0.85**	**0.60**	**5.4E-05**	**2.0E-03**	**0.04**	**0.62**
LOC124904616	**1.16**	**-0.76**	**3.6E-05**	**3.6E-03**	**0.03**	**0.62**
NAT8L	**-1.11**	**0.69**	**3.7E-05**	**6.1E-03**	**0.03**	**0.62**
LMF1	-0.55	0.52	2.8E-04	1.2E-04	0.06	0.62
H2AC21	0.87	-0.89	2.3E-03	4.2E-04	0.11	0.62
LOC124902388	0.83	-0.80	1.8E-03	7.3E-04	0.10	0.62
RNY1	1.23	-1.28	4.4E-03	9.0E-04	0.14	0.62
APC2	-0.92	0.70	2.5E-04	2.4E-03	0.06	0.62
SUCNR1	0.98	-0.82	1.2E-03	2.7E-03	0.09	0.62
H2BC18	0.62	-0.57	3.5E-03	3.0E-03	0.13	0.62
RPS6P1	0.71	-0.65	3.8E-03	3.4E-03	0.14	0.62
H2AC13	1.46	-1.07	2.5E-04	3.5E-03	0.06	0.62
CYSLTR1	0.79	-0.71	3.7E-03	3.6E-03	0.13	0.62
MTATP8P2	-1.00	0.93	4.9E-03	3.6E-03	0.15	0.62
CTNND2	-0.78	0.58	3.9E-04	4.1E-03	0.06	0.62
CYP2A6	-0.92	0.80	3.2E-03	5.0E-03	0.13	0.62
TDRD12	-0.63	0.56	6.4E-03	6.3E-03	0.16	0.62
SLITRK5	-1.28	0.97	1.1E-03	6.7E-03	0.09	0.62
SLC52A3	-0.74	0.66	6.3E-03	6.8E-03	0.16	0.62
H2BC11	0.85	-0.64	1.1E-03	6.9E-03	0.09	0.63
RNF212	1.24	-0.86	3.7E-04	7.1E-03	0.06	0.63
PCDHGA4	-1.10	0.81	9.6E-04	7.7E-03	0.09	0.65
TTC36	-0.64	0.53	4.3E-03	8.1E-03	0.14	0.66
LOC124903420	1.17	-1.01	5.9E-03	8.2E-03	0.15	0.66
**Cluster**	**2**	**3**	**2**	**3**	**2**	**3**
SBF1P1	0.86	-0.92	0.006	0.001	0.78	0.62
RNU6-9	0.60	-0.58	0.004	0.002	0.78	0.62
SLC26A7	1.18	-0.95	0.001	0.003	0.78	0.62
OR10AD1	-0.62	0.57	0.008	0.006	0.78	0.62
